# Antifungal Activity and DNA Topoisomerase Inhibition of Hydrolysable Tannins from *Punica granatum* L.

**DOI:** 10.3390/ijms22084175

**Published:** 2021-04-17

**Authors:** Virginia Brighenti, Ramona Iseppi, Luca Pinzi, Annamaria Mincuzzi, Antonio Ippolito, Patrizia Messi, Simona Marianna Sanzani, Giulio Rastelli, Federica Pellati

**Affiliations:** 1 Department of Life Sciences, University of Modena and Reggio Emilia, Via G. Campi 103/287, 41125 Modena, Italy; virginia.brighenti@unimore.it (V.B.); ramona.iseppi@unimore.it (R.I.); luca.pinzi@unimore.it (L.P.); patrizia.messi@unimore.it (P.M.); 2 Department of Soil, Plant and Food Sciences, University of Bari Aldo Moro, Via Amendola 165/A, 70126 Bari, Italy; annamaria.mincuzzi@uniba.it (A.M.); antonio.ippolito@uniba.it (A.I.); 3 CIHEAM-Bari, Via Ceglie 9, 70010 Valenzano, Italy; sanzani@iamb.it

**Keywords:** *Punica granatum* L., pomegranate, punicalagin, antifungal activity, *Candida albicans*, topoisomerase

## Abstract

*Punica granatum* L. (pomegranate) fruit is known to be an important source of bioactive phenolic compounds belonging to hydrolysable tannins. Pomegranate extracts have shown antifungal activity, but the compounds responsible for this activity and their mechanism/s of action have not been completely elucidated up to now. The aim of the present study was the investigation of the inhibition ability of a selection of pomegranate phenolic compounds (i.e., punicalagin, punicalin, ellagic acid, gallic acid) on both plant and human fungal pathogens. In addition, the biological target of punicalagin was identified here for the first time. The antifungal activity of pomegranate phenolics was evaluated by means of Agar Disk Diffusion Assay and minimum inhibitory concentration (MIC) evaluation. A chemoinformatic analysis predicted for the first time topoisomerases I and II as potential biological targets of punicalagin, and this prediction was confirmed by in vitro inhibition assays. Concerning phytopathogens, all the tested compounds were effective, often similarly to the fungicide imazalil at the label dose. Particularly, punicalagin showed the lowest MIC for *Alternaria alternata* and *Botrytis cinerea*, whereas punicalin was the most active compound in terms of growth control extent. As for human pathogens, punicalagin was the most active compound among the tested ones against *Candida albicans* reference strains, as well as against the clinically isolates. UHPLC coupled with HRMS indicated that *C. albicans*, similarly to the phytopathogen *Coniella granati*, is able to hydrolyze both punicalagin and punicalin as a response to the fungal attack. Punicalagin showed a strong inhibitory activity, with IC_50_ values of 9.0 and 4.6 µM against *C. albicans* topoisomerases I and II, respectively. Altogether, the results provide evidence that punicalagin is a valuable candidate to be further exploited as an antifungal agent in particular against human fungal infections.

## 1. Introduction

It is well-known that natural compounds derived from plants represents a rich source of privileged chemical entities for drug discovery, since they are characterized by favorable chemical properties, a high structural diversity, and positive effects on human health [1,2]. A recent literature review has identified nearly a hundred novel natural products having promising antifungal activity against human pathogens [2]. These compounds originate from a variety of organisms, comprising bacteria, algae, fungi, sponges, and plants [2]. Focusing on natural antifungal compounds derived from plants, previous studies have demonstrated that *Punica granatum* L. (pomegranate) extracts rich in punicalagin present compelling antifungal activity [3,4], even though the mechanism/s of action behind this activity has never been fully elucidated.

In a recent study, pomegranate cultivars with different susceptibility to the fungal etiological agent of fruit dry rot *Coniella granati*, also known as *Pilidiella granati*, were tested for their phenolic profile. The HPLC analyses of the extracts indicated the presence of characteristic peaks in the fungal inoculated samples, corresponding to phenolic compounds derived from the degradation of punicalagin, which is the main ellagitannin present in this fruit [5]. Since *C. granati* is fairly able to enzymatically lysate this class of compounds, it is reasonable that the phenolic production is a response to the pathogen attack, i.e., the fungus hydrolyses them in a positive feedback mechanism that ends up in a minor susceptibility of some varieties [5]. This finding supports also the hypothesis that pomegranate polyphenols might play a role in the different susceptibility of pomegranate cultivars to *C. granati*, their content being a putative marker in selecting programs of new resistant germplasm [5].

**Figure 1:**
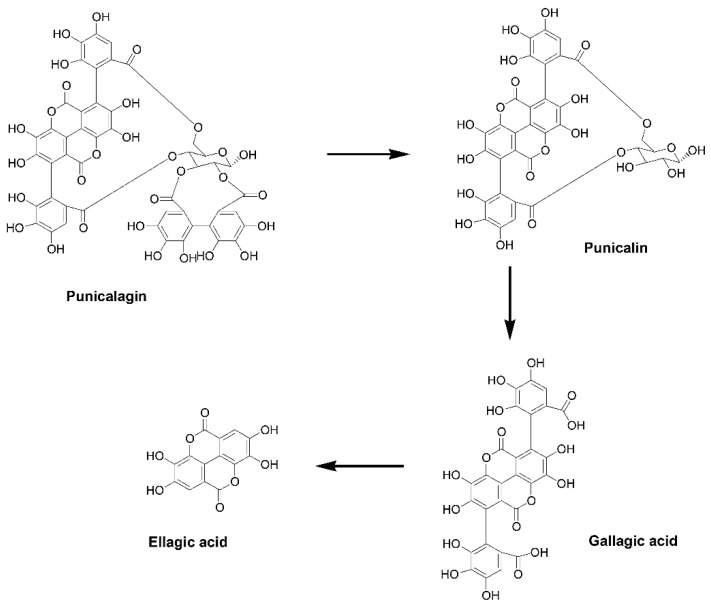
Biodegradation pathway of punicalagin.

Punicalagin, highly present in pomegranate rind, is the putative precursor of punicalin (Figure 1) [6]. Gallagic acid is an intermediate molecule, which in turn is generated after punicalin degradation, and is the precursor of ellagic acid, which is the last product of ellegitannin biodegradation (Figure 1) [6].

The surge in resistance to fungicides among pathogenic populations is an emergent issue in both agriculture and medicine [7], and a clear example of the importance of a One Health approach. As for human pathogenic fungi, several classes of antifungal drugs are in clinical use, but drug-resistant *Candida albicans* and toxicity-related reactions to existing compounds strongly suggest that new therapeutic agents are required [7]. The emergence of fungicide-resistant isolates of human pathogens has been related to the exposure to triazole fungicides used in agro-ecosystems [7]. The low number of antifungal agents in clinical use, as opposed to the large number of agricultural fungicides with similar mode of action, is considered as a risk factor that limits the success of the therapeutic use of existing antifungal drugs [7], again pointing to the need of developing new antifungal agents. In this context, natural compounds derived from plants could represent a promising alternative to synthetic compounds to fight fungal pathogens.

In this work, we evaluated the antifungal activity of phenolic compounds from pomegranate, including punicalagin, punicalin, ellagic acid, and gallic acid, against a wide spectrum of pathogenic fungi, including both fungal phyto- and human pathogens. Chemoinformatic analyses were used to identify potential biological targets of these natural products, suggesting that topoisomerases I and II could be involved in their antifungal activity. In vitro assays confirmed that punicalagin is a potent *C. albicans* topoisomerase inhibitor.

## 2. Results and Discussion

### 2.1. Antifungal Activity of Pomegranate Polyphenols against Fungal Phytopathogens

In a previous study [5], a putative protective effect of pomegranate polyphenols against *C. granati* was observed. To deeper investigate the activity of these compounds against some of the most devastating pomegranate phytopathogens belonging to *Alternaria, Botrytis, Colletotrichum*, and *Coniella* genera, a simple, rapid and reliable spectrophotometric method was used. It allows the monitoring of fungal growth by measuring the turbidity of the culture broth [8]. The results obtained highlighted differences among phytopathogens response to the treatments (Table 1, Figures S1–S4).

As for punicalagin (Figure S1), a reduction of *A. alternata* and *B. cinerea* growth was observed after 18 and 36 h of incubation, respectively. The MIC value for both these fungi was 92.2 µM (Table 1). However, the control extent was lower than that of the fungicide imazalil, used for comparative purposes (positive control) at the label dose. A better performance was observed for *C. acutatum* s.s. and *C. granati*, with a similar trend between them. The MIC value for these fungi was 184.4 µM (Table 1). The control effect remained comparable to that of imazalil until 24 and 48 h of incubation for *C. acutatum s.s.* and *C. granati*, respectively. A 66 and 83% growth reduction was observed at 48 h for *C. acutatum* s.s. and *C. granati*, respectively.

Concerning punicalin (Figure S2), a stronger growth reduction was obtained as compared to punicalagin, although the MIC value for all fungi was 255.6 µM (Table 1). In particular, even if *A. alternata* and *B. cinerea* growth was controlled by the treatment, they were less sensitive than other fungi. On *C. acutatum* s.s., the effect of punicalin was comparable to that of imazalil up to 24 h of incubation, with a reduction by 83%. Similarly, a reduction up to 92% was observed in *C. granati* up to 48 h of incubation, providing the best control activity among tested fungi.

Overall, ellagic acid showed no or little antifungal activity against *B. cinerea* and *A. alternata*, while better results were observed for *C. acutatum* s.s. and particularly for *C. granati* (Figure S3). Furthermore, a dose-effect was recorded with the lowest concentration being the most effective; the MIC value was 165.4 µM for all tested phytopathogens (Table 1). At that concentration, ellagic acid delayed the growth of *C. acutatum* s.s. up to 24 h of incubation, similarly to imazalil. Concerning *C. granati*, a reduction of growth was observed after 18 h of incubation, reaching up to 60% up to 48 h of incubation. Even in this case, the lowest concentration resembled the activity of imazalil up to 48 h of incubation.

Concerning gallic acid, all the tested concentrations provided antifungal activity against *A. alternata* (Figure S4). Particularly, the absorbance (Abs) values for gallic acid and the fungicide imazalil were similar at 12–24 h of incubation. Gallic acid showed a dose-dependent antifungal activity against *B. cinerea*, with the lowest concentration as the most effective (Figure S4). Its performance was comparable to the fungicide control up to 42 h of incubation. In the presence of gallic acid, a similar trend, with a considerable growth reduction up to 48 h of incubation was observed for *C. acutatum* s.s. and *C. granati*, independently from the concentration tested (Figure S4). Furthermore, the effect on *C. granati* was comparable to that of imazalil. The MIC values of gallic acid were 587.8 µM for all phytopathogens (Table 1).

Punicalin and punicalagin provided responses similar to gallic acid, for the fungal groups *A. alternata*–*B. cinerea* and *C. acutatum* s.s.–*C. granati*. Overall, punicalin was the most effective treatment, while ellagic acid was the least effective one, even though this latter was tested at lower concentrations, due to solubility issues. *C. granati* and *B. cinerea* proved to be the most and the least sensitive fungus to treatments, respectively. This was reasonably due to the different ability of the tested phytopathogens to hydrolyze polyphenols [5]. The higher susceptibility of *C. granati* was probably due to its metabolism, since it has been demonstrated that other species of the genus possess enzymes involved in phenolic compound degradation, such as tannases and aryl sulfotransferase [9]. Furthermore, among the tested phytopathogens, *C. granati* is the only one specific to pomegranate, and, as such, reasonably able to withstand its defensive weapons.

In general, the lower concentrations of compounds were the most effective against the pathogens, suggesting a hormetic effect, already observed for other natural compounds [10]. The effect of gallic acid, punicalin and punicalagin on *C. granati* was comparable to that of a conventional fungicide at the label concentration. This is an important result, even if preliminary, considering that there is an increasing search for safer alternatives to fungicides that might have similar performances [10]. Similarly, it has been reported that extracts of grape tendrils containing ellagic acid were effective against *Fusarium* spp., and at higher concentrations, also against *Alternaria solani* and *B. cinerea* [11]. In addition, gallic acid, contained in both finger millet husk and whole flour, has been successfully tested to control *Aspergillus flavus* growth [12]. Finally, pistachio extracts rich in gallic acid were successful in inhibiting *Aspergillus niger* [13]. Polyphenols effect might be ascribed to changes in fungal cell membrane permeability [14], as well as to the induction of host defensive mechanisms [15]. This information might contribute to understand host-pathogen interaction of the fungi causing huge losses in pomegranate worldwide trades [16] as a basis for elaborating eco-friendly control strategies.

### 2.2. Antifungal Activity of Pomegranate Polyphenols against Human Pathogens

#### 2.2.1. Agar Disk Diffusion Assay

In view of the results obtained on phytopathogens, a preliminary screening on the capacity of pomegranate hydrolysable tannins (i.e., punicalagin and punicalin), ellagic acid and gallic acid, to inhibit human fungal pathogens was conducted by means of the Agar Disk Diffusion Assay. The results are shown in Table S1. The inhibition zone of fungal growth ranged from 13 to 23 mm for most of the tested strains at the concentration of 50 µg by using punicalagin, punicalin, and gallic acid. At the concentration of 100 µg, punicalagin showed a good antifungal activity, with range of inhibition zone from 21 to 30 mm for 82% of *Candida* strains and for 63% of other genera tested. Instead, punicalin and gallic acid, at the concentration of 100 µg, inhibited the growth of *Candida* (91% and 73%, respectively) and the other fungi strains (63% and 25%) with a range zone from 11 to 20 mm. At the higher concentration (250 µg), most of the tested strains with punicalagin, punicalin and gallic acid displayed a fungal inhibition zone from 21 to 30 mm. Ellagic acid showed a good anti-candida activity at a concentration of 25 μg; in particular, 36% of *Candida* tested strains displayed a growth inhibition zone in a range from 11 to 20 mm, while 64% from 21 to 30 mm. At a higher concentration of ellagic acid (62.5 µg) most of the strains treated exhibited a range of growth inhibition from 21 to 30 mm (73% for *Candida* and 100% for the other strains) The inhibition zone of the two antifungals agents used as the positive controls (fluconazole and amphotericin B at 25 µg) was from 21 to 30 mm against 73% and 46% of *Candida* strains, respectively. For punicalagin, punicalin and gallic acid a concentration of 100 µg was required to obtain an inhibition zone from 21 to 30 mm, compared to that of 25 µg for the antifungal agents. Instead, ellagic acid at the same concentration of the two antifungals showed a good anti-*Candida* activity.

#### 2.2.2. Minimum Inhibitory Concentration Assessment

The antifungal activity of the target compounds was further confirmed by the determination of their MIC values on all tested strains. Punicalagin displayed the most promising antifungal activity towards the pathogens considered in this work (Table 2). In particular, a good activity was observed for punicalagin especially toward *C. albicans*, in accordance with the literature [17]. Indeed, punicalagin antifungal activity was of the same order of magnitude as amphotericin B against two *C. albicans* reference strains and *C. parapsilopsis*, while it was more effective compared to fluconazole and amphotericin B for two clinically isolates (*C. albicans* 1 and 2), resistant to both antifungals. Punicalin was found to possess a MIC value lower than that of amphotericin B (4.3 µM) against the two clinically isolates *C. albicans* 2 and *C. albicans* H (MIC values of 3.8 µM and 1.9 µM, respectively), which are resistant to both fluconazole and amphotericin B. As for ellagic acid, the best activity was observed toward three resistant *Candida* strains (1, 2, and 44), which were clinically isolated.

#### 2.2.3. Time Kill Curves

**Figure 2:**
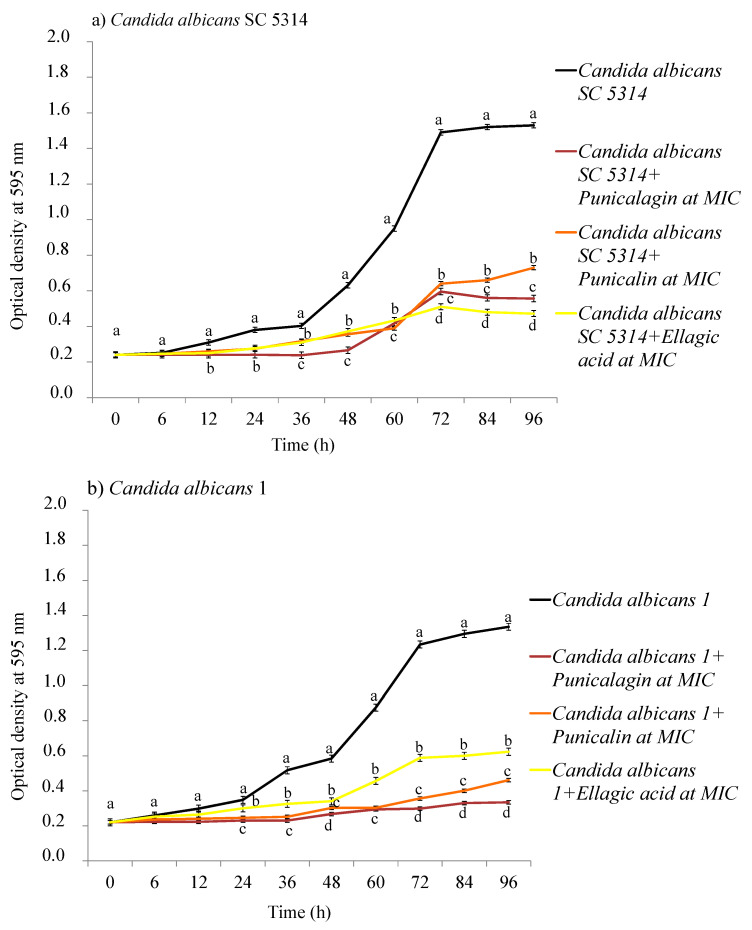
Time-kill curves of punicalagin, punicalin and ellagic acid against *Candida albicans* SC 5314 classified strain (panel (**a**)) and the clinically isolated strain *C. albicans* 1 (panel (**b**)). Results were expressed as mean ± SD of the three determinations. Different letters indicated statistical differences (*p* ≤ 0.05).

A time-course of the susceptibility of the reference strain *C. albicans* SC 5314 and the clinical strain *C. albicans* 1 to the pomegranate compounds, selected on the basis of the diameter of their inhibition zones and their low MIC values, was assessed. A major reduction in the number of *C. albicans* SC 5314 cells, treated with punicalagin at a concentration corresponding to its MIC value, was observed in the first 48 h of the experiment (Figure 2a). Ellagic acid showed better antifungal activity against the reference *C. albicans* strain after 72 h of exposure. Punicalin displayed similar inhibitory activity to ellagic acid in the first 60 h of treatment, after which it exhibited a lower activity if compared with the other two compounds (Figure 2a).

Regarding *C. albicans* 1 strain, punicalin and punicalagin showed a similar inhibitory activity on the fungal growth during the first hours of the experiment; after 60 h of incubation, punicalin becomes less effective than punicalagin, which preserved the antifungal activity until the end of the test (96 h) (Figure 2b). Ellagic acid exhibited the lowest activity compared with the other compounds, although it considerably reduced fungal growth (Figure 2b).

### 2.3. Quantitative Analysis of Pomegranate Polyphenols in Lysates from Candida albicans Cultures

**Figure 3:**
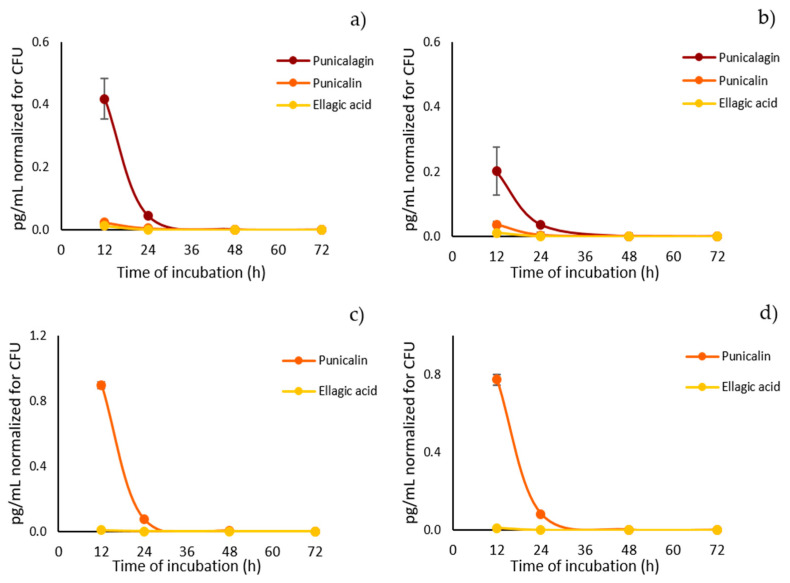
Trend of pomegranate polyphenols in *Candida albicans* SC 5314 and *C. albicans* 1 lysates. *C. albicans* SC 5314 was incubated with punicalagin (panel (**a**)) and punicalin (panel (**c**)); the clinically isolated *C. albicans* 1 was incubated with punicalagin (panel (**b**)) and punicalin (panel (**d**)). The amounts of pomegranate polyphenols detected in cell lysates are expressed as pg/mL and they were normalized for the number of colony forming units (CFU) at the different incubation time.

To assess the ability of *C. albicans* to lysate pomegranate hydrolysable tannins by its enzymatic activity as a response to the pathogen attack, in analogy to what has been previously described for the phytopathogen *C. granati* [5], a method based on ultra high-performance liquid chromatography coupled with high-resolution mass spectrometry (UHPLC-HRMS) was developed and applied for the analysis of polyphenols in lysates from *C. albicans* cultures. Quantitative data related to the amount of pomegranate polyphenols detected in cell lysates from *C. albicans* cultures belonging to two representative strains (one classified and one clinically isolated) treated with punicalagin and punicalin are shown in Table S2. For better data visualization, quantitative data of punicalagin, punicalin and ellagic acid were normalized to the number of colony forming units (CFU) at the different time of incubation, as shown in Figure 3. As it is possible to see from the graphs (Figure 3), the amount of punicalagin in both *C. albicans* strains dramatically decreases between 12 and 24 h of incubation (Figure 3a,b). The same trend can be observed for *C. albicans* incubated with punicalin (Figure 3c,d). In particular, when *C. albicans* strains were incubated with punicalagin, a significant amount of punicalin was detected already after 12 h of incubation, which steeply decreased after 24 h of incubation, reflecting the same trend as its precursor. The presence of ellagic acid was already detected after 12 h when *C. albicans* strains were incubated with both punicalagin and punicalin, and its amount did not vary as a function of the incubation time, as it remained approximately constant.

These data suggest a rapid lysis of punicalagin and punicalin by fungal enzymes, as their concentration significantly decreased after only 24 h of incubation. Punicalin is one of punicalagin lysis products, as it was detected in samples incubated with punicalagin, though punicalin itself is further rapidly broken down into smaller molecules. A rapid breakdown of punicalin into smaller molecules could explain the observed decrease of its concentration in the lysates. Interestingly, indeed, the amount of both punicalagin and punicalin in culture mixtures seems to follow the same trend over time, thus suggesting a common lysis process by *C. albicans* enzymes. Gallic acid was not detected under the applied conditions in the HPLC-HRMS chromatograms. Ellagic acid is probably constantly produced by the lysis of both punicalagin and punicalin, since the beginning of the incubation

### 2.4. Identification of Topoisomerase I and II as Potential Puncicalagin Targets from Chemoinformatic Analysis

To identify potential biological targets of punicalagin (CHEMBL ID: CHEMBL506814), which resulted the most active compound in particular against human fungal pathogens, a series of ligand-based similarity analyses were performed within the ChEMBL database [18]. Similarity calculations were restricted to punicalagin, which represents the most abundant and active compound among the investigated ones, and it is also the precursor of both punicalin and ellagic acid [19]. The similarity profile of punicalagin was evaluated with respect to the ChEMBL ligands, by using the ROCS software [20,21]. This approach has already demonstrated to provide valuable results in previous in silico repositioning studies, also related to natural products [22,23,24,25]. The ChEMBL database was selected among those publicly available, because it provides chemical, structural and bioactivity annotations of about 1.9 million compounds on around 13,000 different biological targets. Therefore, it represents a valuable source of information for in silico repositioning and target identification tasks. Compounds extracted from ChEMBL were first prepared for the 3D ligand-based similarity analyses. Considering that ligand-based similarity estimations were performed using 3D conformations, molecular dynamics simulations in explicit solvent were performed to obtain the most energetically stable conformation(s), for each protonation state of punicalagin present at physiological pH, as evidenced by the measured pK_a_ values (see “Materials and Methods” section for the experimental details). These conformations turned out to be quite stable over time, with average Root Mean Square Deviations (RMSD) of only 0.86 ± 0.19 Å and 0.74 ± 0.14 Å for punicalagin 3O (dissociated 3-hydroxyl) and 8O (dissociated 8-hydroxyl) protonation states, respectively. Figure S5a,b show the punicalagin 3O and 8O representative conformations selected as queries for the 3D-ligand-based analyses, respectively.

Afterwards, all ChEMBL compounds were subjected to a series of 3D ligand-based similarity analyses against the selected punicalagin queries. The obtained ligand alignments were then ranked according to their Tanimoto Combo similarity scores and duplicates deriving by comparisons of the same ChEMBL ligand towards different protonation states of punicalagin were removed. This allowed us to retain the ligand-alignments with the best similarity scores, regardless of the protonation state of the query. Cut-off values on the Tanimoto Combo similarity scores were not used during the 3D ligand-based estimations, but visual inspection was limited to the first 100 top-ranking alignments. The performed ligand-based analyses allowed us to predict and rank the potential targets of punicalagin according to the degree of 3D similarity of ChEMBL ligands with punicalagin. Interestingly, the top-scoring similarity list was highly populated by known ligands of DNA topoisomerases I and II, which ranked at the first positions of the top-scoring alignments (Table 3), suggesting that these two enzymes may be putative punicalagin targets. The top-scoring ligands included chebulagic acid, corilagin, several anthracyclines and epipodophyllotoxine derivatives, i.e., compounds with proven topoisomerases I and II mechanisms of action.

**Figure 4:**
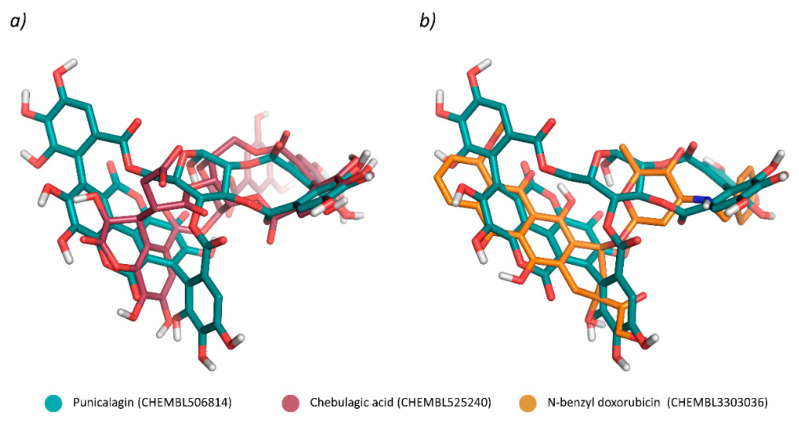
Ligand-based alignments of punicalagin with the CHEMBL525240 (chebulagic acid) and CHEMBL3303036 (N-benzyl doxorubicin) compounds, predicted with ROCS. (Panel (**a**)) shows the structures of punicalagin (dark teal sticks) and CHEMBL525240 (raspberry sticks) providing the best shape-based alignment. (Panel (**b**)) shows the shape-based alignment obtained for punicalagin (dark teal sticks) with the CHEMBL3303036 (orange sticks) compound. The image was created with PyMol (The PyMOL Molecular Graphics System, Version 1.8, Schrödinger, LLC).

In particular, punicalagin provided good structural overlaps with chebulagic acid (ChEMBL ID: CHEMBL525240, rank #8) (Figure 4a), a known inhibitor of DNA topoisomerase I, with reported IC_50_ of 50 nM [26]. Moreover, similarities were also observed between punicalagin and corilagin (CHEMBL ID: CHEMBL449392, rank #13), another DNA topoisomerase I ligand [26]. Significant similarities were also noted between punicalagin and a number of anthracycline analogues, such as N-benzyl doxorubicin (ChEMBL ID: CHEMBL3303036, rank #65) (Figure 4b), N,N-dibenzyl doxorubicin (ChEMBL ID: CHEMBL3248005, rank #14), a disaccharide derivative of daunorubicin (ChEMBL ID: CHEMBL2367695, rank #19) and a hydrazone derivative of N-morpholin-doxorubicin (ChEMBL ID: CHEMBL66563, rank #100), indicating a potential DNA topoisomerase II inhibitory activity [27,28]. Finally, good overlaps were observed between punicalagin and CHEMBL3974286 (rank #99), a norcantharadin drug conjugate of 4′-demethylepipodophyllotoxin, and with the 5-FU drug conjugate CHEMBL362359 (rank #41) structurally related to the DNA topoisomerase II drug etoposide [29]. Likewise, similarity with the two 4-acetic acid ester derivatives of podophyllotoxin CHEMBL4092572 (rank #48) and CHEMBL4060624 (rank #92) further corroborated potential topoisomerase II activity.

According to the obtained alignments, the two gallate groups at the dioxacyclododecine-dione moiety of chebulagic acid perfectly overlapped with those in the dioxecine-dione macrocycle of punicalagin. The acetyl-trihydroxy-chroman-one group of chebulagic acid only partially superimposed with the ellagic moiety of punicalagin in the predicted alignment, due the different size of these chemical moieties. Worth of note, the 3,4,5-trihydroxybenzene group at the position 5 of chebulagic acid did not overlap with any group of punicalagin, probably because of conformational restraints on the predicted alignment. Regarding the CHEMBL3303036 compound (N-benzyl doxorubicin), the N-benzyl moiety partially overlapped with one of the two distal gallate groups of punicalagin, while the naphthacenequinone portion superimposed with the acidic ellagic moiety of punicalagin. In this case, the overlap between these moieties is mainly driven by the high degree of shape similarity.

Based on these predictions, the possibility that punicalagin may exert antifungal activity through inhibition of yeast DNA topoisomerases represents an important and hitherto unexplored mechanism of action of this natural compound. Indeed, while topoisomerases are well-known and exploited targets in anticancer drug discovery, their relevance and involvement as antifungal drug targets has far less been investigated [30,31]. Recent studies demonstrated that inhibitors, such as idarubicin and doxorubicin (the parent compound of CHEMBL3303036), possess antifungal activity against different genera of fungi [32], further supporting the selection of punicalagin as a potential target of fungal DNA topoisomerases I and II. However, to the best of our knowledge, none of the antifungal drugs in clinical use act through inhibition of topoisomerases. To confirm our predictions, punicalagin and related degradation products were *in vitro* tested against yeast DNA topoisomerases I and II.

### 2.5. In Vitro Interactions of Pomegranate Polyphenols with Candida Topoisomerases

The IC_50_ values of pomegranate compounds as inhibitors of yeast topoisomerase I and II enzymes were determined in a concentration range 0.001–200 μM, and the results are shown in Table 4. All the compounds were able to inhibit the catalytic activity of both topoisomerase I and II, inducing inhibition of the relaxation of supercoiled DNA.

As shown in Table 4, punicalagin turned out to be the most effective molecule among the tested ones, with IC_50_ values of 9.0 and 4.6 µM against yeast topoisomerases I and II, respectively. Remarkably, punicalagin resulted also more potent than the reference inhibitors camptothecin (topo I inhibitor, IC_50_ of 17.8 μM) and etoposide (topo II inhibitor, IC_50_ of 15.9 μM), while punicalin inhibited topoisomerases I and II with IC_50_ values of 14.7 µM and 40.7 µM, respectively. Ellagic acid had the lowest activity, the experimentally estimated IC_50_ values being 56.6 and 53.9 µM for topoisomerases I and II, respectively (Table 4).

These results confirm that punicalagin is a potent fungal topoisomerase I and II inhibitor and it might represent a novel therapeutic agent to be further exploited. By taking into account the previously described capacity of punicalagin to inhibit squalene epoxidase [33], which is involved in ergosterol biosynthesis pathway, it is plausible to consider this compound as a promising antifungal agent with a multi-target inhibition mechanism.

Chemoinformatic analyses were also performed to evaluate whether punicalagin, punicalin and ellagic acid might be pan-assay interference compounds (PAINS) [34], thus potentially providing false positive readouts in the in vitro assays. In particular, in silico investigations were performed by using the FILTER software (OpenEye, version 3.1.2.2) [35], which allows to detect PAINS, as defined by Baell et al. [34]. The compounds were classified to be potential PAINS based only on the presence of catechol and ester moieties. However, one should note that these chemical moieties are very often present in drugs and clinically safe candidates, and that these filters were initially developed for synthetic small molecules and not natural compounds [34]. Applying PAINS rules to natural compounds may result challenging [36], due to their different origin and structural complexity. Further efforts were also addressed to evaluate whether the investigated compounds were listed among the Invalid Metabolic Panaceas (IMPs) ligands defined by Bisson et al. [37], i.e., natural products potentially subjected to spurious bioassay interferences and with a high number of reported bioactivities, classifiable as PAINS. Interestingly, punicalagin, punicalin and ellagic acid were not classified as potential IMPs, further supporting their validation as inhibitors of topoisomerase I and II enzymes.

## 3. Materials and Methods

### 3.1. Chemicals and Reagents

Reference compounds of punicalagin, punicalin, ellagic acid, and gallic acid (purity 98%) were purchased from Aktin Chemicals Inc. (Chendgu, China). Camptothecin and etoposide were acquired from TCI Europe N.V. (Zwijndrecht, Belgium). Glycerol was from Incofar (Modena, Italy). Fluconazole (≥98%), amphotericin B (≥80%), Tris-HCl, ethylenediaminotetraacetic acid (EDTA), sodium chloride (NaCl), bovine serum albumin (BSA), spermidine, sucrose, bromophenol blue, tris acetate, chloroform, isoamyl alcohol, dimethyl sulfoxide (DMSO), methanol (MeOH), formic acid (HCOOH), Tween-80, lysozyme, sodium dodecyl sulfate (SDS) and imazalil were from Sigma-Aldrich, Merck KGaA (Darmstadt, Germany). Sabouraud Dextrose broth (SDB), Sabouraud Dextrose agar (SDA), Sabouraud Dextrose agar with Chloramphenicol 50 mg (SDA-CAF) and potato dextrose agar (PDA) were purchased from Oxoid Limited (Basingstoke, Hampshire, UK). CHROMID agar (CHROMID^®^ Candida^®^) was from bioMérieux (Milan, Italy). Water (H_2_O) was purified by using a Milli-Q Plus185 system from Millipore (Milford, MA, USA).

### 3.2. Pomegranate Fungal Pathogens Strains

*Alternaria alternata* (Fr.) Keissl. (strain AL19), *Coniella granati* (syn. *Pilidiella granati*) (Sacc.) Petr. & Syd (strain M0_C2), *Botrytis cinerea* Pers. (strain B2) and *Colletotrichum acutatum sensu stricto* (s.s., Simmonds) (strain M146-2), isolated from naturally infected pomegranates, came from the Fungal Collection of Department of Soil, Plant, and Food Sciences, University of Bari Aldo Moro (Bari, Italy).

### 3.3. Human Fungal Pathogens

The antifungal activity of pomegranate compounds was evaluated against 19 clinically isolated fungal strains, including nine belonging to *Candida albicans* species, two belonging to *Aspergillus* genus, one belonging to *Cryptococcus* genus, and one *Saccharomyces cerevisiae*. The *C. albicans* strains were isolated on CHROMID agar, *Aspergillus* and *Cryptococcus* genus were isolated on SDA-CAF and one *Saccharomyces cerevisiae* on SDA. All the isolates were confirmed by matrix-assisted laser desorption ionization (MALDI) time-of-flight mass spectrometry (TOF/MS). Strains from the American Type Culture Collection (ATCC) were included. All strains were maintained in SDB with 20% (*w/v*) glycerol at −80 °C until use and sub-cultured in SDA before each test.

### 3.4. Determination of the Antifungal Activity against Phytopathogens

The above-mentioned phenolic compounds from pomegranate, i.e., punicalagin and punicalin, ellagic acid and gallic acid, were tested against four relevant fungal pathogens of pomegranate fruit. To produce inocula, strains were grown on PDA plates at 24 ± 1 °C in the dark for 7 days. Plates were flooded by 0.05% Tween-80 solution and conidia were dislodged by a sterile spatula. The resulting suspension was counted using a Thoma counting chamber (VWR International, Radnor, PA, USA) and diluted by sterile distilled water to a concentration of 104 conidia/mL.

According to the different solubility of each compound as reported by the manufacturer and relevant bibliography [38], they were initially dissolved in DMSO to obtain stock solutions. Then, these latter were aseptically diluted in sterile distilled water to working concentrations of 1000, 2000, and 4000 µg/mL for gallic acid/punicalin/punicalagin, whereas ellagic acid was tested at 500, 1000, and 2000 µg/mL. To assess the antifungal activity of the selected polyphenolic compounds, micro-spectrophotometric assays were arranged according to Broekaert et al. [8]. PDB medium was prepared according to producer recommendation, and 160 μL were poured in the wells of a 96-well clear flat bottom untreated cell culture plate (Falcon-Corning, Tewksbury, MA, USA). Then, 20 μL of each tested compound and concentration (final concentrations of 100, 200, and 400 µg/mL for gallic acid/punicalin/punicalagin, and 50, 100, and 200 µg/mL for ellagic acid), and 20 μL of conidial suspension (final concentration 2 × 10^2^ conidia/mL) were added. The fungicide imazalil and sterile H_2_O were used as positive and negative control, respectively. Three replicates were set for each combination of substance, concentration and pathogen. Microplates were incubated at 24 ± 1 °C in the dark. Fungal growth was monitored for 72 h measuring the absorbance (Abs) at 405 nm of the micro-cultures using a microplate reader (Multiskan EX, LabSystems Italia s.r.l., Abbiategrasso, Milan, Italy). The MIC, defined as the lowest concentration of the compounds that inhibits visible growth of the tested strains, was calculated. The experiment was performed in triplicate.

### 3.5. Determination of Antifungal Activity against Human Pathogens

#### 3.5.1. Agar Disk Diffusion Assay

The preliminary determination of the antifungal activity of the target compounds (punicalagin, punicalin, ellagic acid, and gallic acid) from pomegranate was carried out by means of the agar disk diffusion assay, according to the standard procedure of the Clinical and Laboratory Standards Institute [39], with slight modifications. Plates containing SDA were uniformly spread with 100 μL of 10^3^ conidia/mL of each strain suspension. Then, 6 mm diameter sterile disks containing three different concentrations of tested compounds (5, 10, and 20 μL of a 10 mg/mL solution of punicalin, punicalagin, and gallic acid and of a 2.5 mg/mL solution of ellagic acid) were placed on the agar surface. Disks containing fluconazole (25 μg), amphotericin B (25 μg) and DMSO (20 μL) were used as the positive and the negative control, respectively. The antifungal activity of pomegranate compounds was evaluated by measuring the diameter of the clear inhibition zone formed right around the disks after 48 h incubation at 30 ± 1 °C. The experiment was carried out in triplicate.

#### 3.5.2. MIC Determination

The MIC values of the tested compounds were determined by broth microdilution methods based on the Clinical and Laboratory Standards Institute (CLSI) reference protocol [40]. The assay was performed in sterile 96-well microplates by dispensing into each well 90 µL of SDB and 10 µL of strains suspensions, to final inoculums concentrations of 10^3^ conidia/mL. Then, 100 µL of serial dilutions of pomegranate phenolics were added in order to reach final concentration in the range 1000 to 0.25 µg/mL. A well containing only 190 µL of SDB and 10 µL of fungi strain, thus without any compound, was used as the negative control, while fluconazole and amphotericin B were used as the positive controls. The plates were incubated at 30 ± 1 °C for 48 h, with an oscillating speed of 150 rpm. The MIC is defined as the lowest concentration of the compounds that inhibits visible growth of the tested strains after the optical density (OD) measured at 570 nm, using a microtiter plate reader (Sunrise™, Tecan Trading AG, Männedorf, Switzerland). All the experiments were repeated three times.

#### 3.5.3. Time-Kill Curves

The growth of both one reference and one clinically isolated strain of *C. albicans* (i.e., *C. albicans* SC 5314 and *C. albicans* 1, respectively) incubated with pomegranate selected compounds (punicalagin, punicalin, ellagic acid, and gallic acid) was evaluated by calculating the change in the optical density of cells. In a 96-well sterile microplate, 90 µL of sterile nutrient broth and 10 µL of the strains were placed in each well from a stock, previously diluted to obtain a density of about 10^3^ conidia/mL. Punicalin, punicalagin, gallic acid, and ellagic acid were added at different concentrations for each well, depending on their MIC value. The microplate was incubated at 30 ± 1 °C with an oscillating speed of 150 rpm, and the optical density (OD) was determined at 595 nm at fixed time intervals (0, 6, 12, 24, 36, 48, 60, 72, 84, and 96 h) of exposure, using an automatic micro plate reader (Sunrise™, Tecan Trading AG, Männedorf, Switzerland). The experiments were replicated three times.

### 3.6. Incubation of Candida albicans Strains with Pomegranate Compounds and Cell Lysis

Two *C. albicans* strains (i.e., *C. albicans* SC5314 and *C. albicans* 1) were incubated with either punicalagin or punicalin for 12, 24, 48, and 72 h in order to assess whether a break of the two tannins into smaller molecules by the microorganism would occur, as previously described [5].

After incubation with the tested compounds, cultures were submitted to cell lysis. In particular, cultures of *C. albicans* in SDB were suspended in PBS. Aliquots of 1 mL were combined with 350 µL of lysozyme, 350 µL of SDS and incubated for 5 min at 70 °C. Then, samples were combined with 300 μL of 96–100% EtOH and centrifuged for 3 min at 15,000 g at room temperature, followed by 0.22 μm membrane filtration [41]. *C. albicans* cells lysates were finally diluted 1:5 (*v/v*) with the initial mobile phase prior the injection into the HPLC system for the quantitative analysis of punicalagin, punicalin, ellagic acid, and gallic acid.

### 3.7. Quantitative HPLC-HRMS Analysis of Pomegranate Compounds in Lysates from Candida albicans Cultures

Quantitative analysis of punicalagin, punicalin, gallagic acid, ellagic acid, and gallic acid in cell lysates from the two above mentioned *C. albicans* strains was performed by means of UHPLC-HRMS. The analyses were carried out on a Thermo Fisher Scientific Ultimate 3000 (Thermo Fisher Scientific, Waltham, MA, USA), equipped with a vacuum degasser, a binary pump, a thermostated autosampler, a thermostated column compartment and a Q-Exactive Orbitrap mass spectrometer with a heated electrospray ionization (ESI) source. The separation of the target analytes was achieved on an Ascentis Express C_18_ column (150 × 3.0 mm I.D., 2.7 µm, Supelco, Bellefonte, PA, USA), as previously described [42]. The mobile phase was composed of (A) 2% HCOOH in H_2_O and (B) 0.5% HCOOH in MeOH-H_2_O (9:1, *v/v*). The analysis was performed under the following gradient: 0–13 min 2% B, 13–18 min from 2 to 5% B, 18–23 min from 5 to 10% B, 23–43 min from 10 to 25% B, 43–53 min from 25 to 50% B, 53–58 min from 50 to 100% B, 58–68 min 100% B, 68–71 min from 100 to 2% B. The post-running time was 5 min. The flow-rate was 0.4 mL/min. The column temperature was set at 30 °C. The sample injection volume was 3 µL. As to the MS detector, the source parameters were set as follows: Capillary temperature 320 °C, vaporizer temperature 280 °C and electrospray voltage ─3.8 kV. The control of the online analyses was carried out using Xcalibur 3.0 software (Thermo Fisher Scientific, San Jose, CA, USA). The analyses were acquired in the full mass data-dependent (FM-dd-MS^2^) and targeted SIM data-dependent acquisition (tSIM-dd-MS^2^) in the negative ion mode at a resolving power of 35,000 full width at half maximum (FWHM). The other mass analyzer parameters were set as follows: Scan range m/z 150–2000, automatic grain control (AGC) target 1 × 10^6^ ions in the Orbitrap analyzer, ion injection time 120 ms and isolation window for the filtration of the precursor ions m/z 1.0. The fragmentation of precursor ions was performed at 20, 30, and 50 as normalized collision energies (NCE). Detection was based on calculated [M−H]^−^ or [M−2H]^2−^ for punicalagin precursor ions with an accuracy of 2 ppm. In particular, punicalagin was detected in the *m/z* range 540.2760–542.2760; punicalin was detected in the *m/z* range 780.3030–782.3030; detection of gallagic acid was performed in the *m/z* range 636.2607–638.2607; ellagic acid and gallic acid were detected in the *m/z* range 300.2490–302.2490 and 168.2643–170.2643.

Stock solution of punicalagin (2.0 µM), punicalin (2.3 µM) and ellagic acid (4.0 µM) were properly diluted with the initial mobile phase to obtain standard calibration solutions. Calibration curves for target compounds were constructed at five calibration levels by plotting the peak areas of the analytes vs. their concentration.

### 3.8. In Silico Ligand-Based Analysis

Compounds with activity annotations expressed as a mean of IC_50_, K_i_, K_d_, EC_50_, ED_50_, GI_50_ and Potency were first downloaded from the ChEMBL database [18,43], for a total of 1,036,633 unique compounds. Then, the compounds were pre-processed with the *LigPrep* (Schrödinger) [44] utility to generate every possible stereoisomer for the ligands with undefined chiral centers, and tautomerization and protonation states at physiological pH. Ligands were also minimized at this stage of the database preparation. Afterwards, the pre-processed ligands were subjected to conformational sampling with the OMEGA software (OpenEye-version 3.1.2.2) [35,45]. Default settings were used in this latter phase of compounds preparation, except for the maximum number of conformers to be generated per ligand, which was set equal to 400. This allowed to exhaustively sample the accessible ligand conformational space.

A different workflow was applied for the preparation of punicalagin. In fact, this compound is a polyphenol with high molecular complexity and different protonation states at physiological pH. To identify an energetically-stable conformation of punicalagin, molecular dynamics (MD) simulations were performed in explicit solvent with the *pmemd.cuda* package available in Amber 18 [46]. In particular, punicalagin was firstly drawn in its most likely protonation states at physiological pH, i.e., those deprotonated at the 3-hydroxyl (punicalagin 3O) and 8-hydroxyl (punicalagin 8O) groups of the ellagic acid moiety (Figure S6) [47,48,49]. Then, molecular dynamics simulations were performed as described below. Punicalagin was prepared by using the Antechamber module available in AmberTools18, the atom types and charges being described according to the GAFF force field and AM1-BCC method, respectively [43,50]. Subsequently, the ligand was solvated in a periodic box of TIP3P water molecules extending 10 Å in each direction and neutralized with chloride ions. Afterwards, the system was minimized by means of 500 steps of the steepest descent and conjugate gradient energy minimization, without constraints. The minimized systems were heated from 0 to 300 K, with 100 ps of constant volume Langevin MD and a collision frequency of 2.0 ps^−1^. A harmonic restraint equal to 5.0 Kcal/mol was applied to the ligand during this phase of the MDs preparation. Then, the system was equilibrated turning to constant pressure, till the obtainment of system convergency. Finally, 200 ns of MD production was performed by using the *pmemd.cuda* utility and the collected trajectories were analyzed with the *CPPTRAJ* module available in AmberTools18 [46]. After equilibration, the conformation of punicalagin turned out to be quite stable over time, and a minimized averaged structure (one for each deprotonation state) was selected as a query for the ligand-based similarity analyses.

The 3D ligand-based similarity analyses were performed with ROCS (OpenEye-version 3.3.2.2) [20,21]. Default settings were used in the similarity assessments, except for the generation of the ligands-alignments, in which the initial step started from 20 random positions. Moreover, the superimposition of the ligands was performed considering also the “color” force field gradients in the overlay optimization step. Finally, the activity annotations of the resulting top-ranking ChEMBL compounds were carefully analyzed to identify potential targets of punicalagin.

In silico investigations were performed by using the FILTER software (OpenEye-version 3.3.2.2) [35], with the implemented “PAINS” filtering settings. The results of this analysis were compared with literature data.

### 3.9. Evaluation of the In Vitro Interactions with Candida Topoisomerases

Fungal topoisomerase I was purified from *C. albicans* strain ATCC 10231, as described by Jiang et al. [51]. Topo I drug kit was purchased from Inspiralis Limited (Norwich, UK). *C. albicans* topoisomerase II was supplied with Topo II drug kit (Inspiralis Limited, Norwich, UK). A supercoiled plasmid DNA (pBR322), which is relaxed by the enzymes with a high affinity for topoisomerase I and topoisomerase II, was used as the substrate. Enzyme activity was assayed in a total volume of 30 μL (250 ng of DNA, test compound, 1 U of purified enzyme, 10 mM Tris-HCl (pH 7.9), 1 mM EDTA, 0.15 M NaCl, 0.1% bovine serum albumin, 0.1 mM spermidine and 5% glycerol) by incubating at 30 °C for 30 min. The reactions were stopped by adding STEB 40% (*w/v*) sucrose, 100 mM Tris-HCl (pH 8), 10 mM EDTA, 0.5 mg/ml Bromophenol Blue and chloroform/isoamyl alcohol (24:1, *v/v*). The extracted DNA was analyzed by electrophoresis on 1% agarose gel in TAE buffer (40 mM Tris acetate, 2 mM EDTA pH 8.5). The gel was stained with ethidium bromide, destained in H_2_O, and photographed on a UV transilluminator (Biosigma S.P.A, Venice, Italy). The intensity of the supercoiled band in each track was determined by scanning the gel image and the enzyme activity was calculated as a percentage of a substrate DNA converted to product. Camptothecin and etoposide were used as the positive controls for their recognized activity on topoisomerase I and topoisomerase II, respectively. The concentration of test compound that prevented 50% of the substrate from being converted into the product (IC_50_) was calculated.

### 3.10. Statistical Analysis

Concerning human fungal pathogens, the statistical analysis was performed by the ANOVA test, using statistical program IBM SPSS Statistics (IBM, Milan, Italy). If statistical analysis determined homogeneity of variances, data from repeated experiments were combined. As for fungal phytopathogens significant differences (*p* ≤ 0.05) were identified by the General Linear Model (GLM) procedure using the Duncan’s Multiple Range Test (DMRT).

## 4. Conclusions

By taking into account the possible emerging correlations between human resistance to antifungal drugs and the widespread use of fungicides in agriculture, pomegranate phenolic compounds were assessed in this study for the control of both fungal diseases of plants and human mycoses in a One Health approach. All the tested phenolic compounds from pomegranate were effective against phytopathogenic fungi. Punicalagin showed the lowest MIC for *A. alternata* and *B. cinerea*, whereas punicalin resulted as the most effective compound in terms of growth control extent. *C. granati* and *B. cinerea* were the most and least sensitive pathogens, respectively. The obtained results might be useful in the understanding of host-pathogen interaction, as well as in the compound exploitation for disease control purposes in agriculture.

As for human pathogenic fungi, punicalagin was found to be the most active compound among the tested ones against *C. albicans* reference strains and also against the clinically isolates. Topoisomerases I and II were identified for the first time by means of a chemoinformatic analysis as the potential biological targets of punicalagin, as confirmed by subsequent *in vitro* inhibition assays. Therefore, punicalagin can be considered as a new candidate to be taken into consideration in clinical practice for its antifungal activity.

## Figures and Tables

**Table 1:** Minimum inhibitory concentration (MIC) of punicalagin, punicalin, ellagic acid, gallic acid and imazalil (fungicide positive control). Data are expressed as μM.

Strain	Punicalagin ^a^	Punicalin ^b^	Ellagic Acid ^c^	Gallic Acid ^d^	Imazalil ^e^
*Alternaria alternata* AL19	92.2	255.6	165.4	587.8	0.3
*Botrytis cinerea* B2	92.2	255.6	165.4	587.8	0.3
*Colletotrichum acutatum s.s.* M146-2	184.4	255.6	165.4	587.8	0.3
*Coniella granati* M0_C2	184.4	255.6	165.4	587.8	0.3

Tested concentrations: ^a^ 92.2–368.8 µM; ^b^ 127.8–511.2 µM; ^c^ 165.4–661.8 µM; ^d^ 587.8–2351.3 µM; ^e^ Label dose.

**Table 2:** Minimum inhibitory concentration (MIC) of punicalagin, punicalin, ellagic acid, gallic acid, fluconazole, and amphotericin B (antifugal positive controls). Data are expressed as μM.

Strain	Punicalagin ^c^	Punicalin ^d^	Ellagic Acid ^e^	Gallic Acid ^f^	Fluconazole ^g^	Amphotericin B ^h^
*Candida albicans* ATCC 10321 ^a^	0.7	127.0	2.5	293.9	40.8	0.1
*Candida albicans* SC 5314 ^a^	0.7	127.0	2.5	293.9	9.8	0.1
*Candida albicans* 1 ^a,b^	1.4	3.8	5.0	293.9	81.6	2.2
*Candida albicans* 2 ^a,b^	1.4	3.8	2.5	293.9	163.2	4.3
*Candida albicans* H ^a,b^	92.2	1.9	331	587.8	81.6	4.3
*Candida albicans* 40 ^a^	5.5	127.0	331	587.8	163.2	0.3
*Candida albicans* 41 ^a^	92.2	127.0	331	293.9	81.6	0.3
*Candida albicans* 44 ^a,b^	92.2	127.0	2.5	293.9	81.6	2.2
*Candida parapsilosis*	0.7	127.0	662	293.9	9.8	0.1
*Candida parapsilosis* 7 ^a^	5.5	3.8	331	587.8	19.6	0.3
*Candida zeylanoides* 33 ^a^	92.2	127.0	331	587.8	19.6	0.1
*Saccharomyces cerevisiae* 42 ^b^	1.4	3.8	662	587.8	4.9	1.1
*Aspergillus brasiliensis* ATCC 16404	2.8	1.9	662	8.8	4.9	0.1
*Aspergillus candidus* 3	92.2	127.0	662	17.6	9.8	0.3
*Aspergillus candidus* 25	92.2	127.0	662	17.6	9.8	0.3
*Cryptococcus neoformans* B 3501	46.1	1.9	331	4.4	4.9	0.1
*Cryptococcus neoformans* ATCC 11240	23.0	1.9	331	4.4	4.9	0.1
*Cryptococcus* 67	92.2	127.0	662	8.8	9.8	0.1
*Cryptococcus* var. grubii H99 serotype A	92.2	127.0	662	4.4	4.9	0.1

^a^ Strains resistant to fluconazole. ^b^ Strains resistant to amphotericin B. Tested concentrations: ^c^ 0.35–921.9 µM; ^d^ 0.48–1270 µM; ^e^ 1.24–3309 µM; ^f^ 2.2–5878 µM; ^g^ 1.22–326.5 µM; ^h^ 0.03–27.1 µM.

**Table 3:** ChEMBL ligands related to DNA topoisomerases I and II identified within the first 100 top-ranking alignments of the ChEMBL/Punicalagin 3D ligand-based similarity comparisons.

Reference	Compound Name	ChEMBL ID	Rank #
Punicalagin	Chebulagic acid	CHEMBL525240	8
Punicalagin	Corilagin	CHEMBL449392	13
Punicalagin	N, N-dibenzyl doxorubicin	CHEMBL3248005	14
Punicalagin	Disaccharide derivative of daunorubicin	CHEMBL2367695	19
Punicalagin	4β-5-FU-substituted 4′-demethylepipodophyllotoxin	CHEMBL362359	41
Punicalagin	4-Acetic acid ester derivative of podophyllotoxin	CHEMBL4092572	48
Punicalagin	N-benzyl doxorubicin	CHEMBL3303036	65
Punicalagin	4-Acetic acid ester derivative of podophyllotoxin	CHEMBL4060624	92
Punicalagin	4β-Norcantharidin-substituted -4′-demethylepipodophyllotoxin	CHEMBL3974286	99
Punicalagin	Hydrazone derivative of N-morpholin-doxorubicin	CHEMBL66563	100

**Table 4:** Activity of punicalagin, punicalin, ellagic acid, and reference compounds on topoisomerase I and topoisomerase II from *Candida albicans*. The IC_50_ values are expressed as mean ± SD (µM).

Compounds	IC_50_ Value (µM)	
	Topoisomerase I	Topoisomerase II
Punicalagin	9.0 ± 1.2	4.6 ± 0.4
Punicalin	14.7 ± 0.9	40.7 ± 0.8
Ellagic acid	56.6 ± 1.2	53.9 ± 0.2
Camptothecin	17.8 ± 0.7	-
Etoposide	-	15.9 ± 0.7

## Data Availability

Not acceptable.

## Supplementary Materials

The following are available online at https://www.mdpi.com/article/10.3390/ijms22084175/s1, Figure S1: Growth of *Alternaria alternata* (panel a), *Botrytis cinerea* (panel b), *Colletotrichum acutatum sensu stricto* (*s.s.*) (panel c) and *Coniella granati* (panel d) for 72 h at 24 (±1) °C in the dark in presence of different concentrations of punicalagin (PG100, PG200, PG400 mg/mL). H_2_O and imazalil were the negative and positive control, respectively. For each pathogen and time point, results are the mean of three replicate values ± standard deviation (SD), Figure S2: Growth of *Alternaria alternata* (panel a), *Botrytis cinerea* (panel b), *Colletotrichum acutatum sensu stricto* (*s.s.*) (panel c) and *Coniella granati* (panel d) for 72 h at 24 (±1) °C in the dark in presence of different concentrations of punicalin (PA100, PA200, PA400 mg/mL). H_2_O and imazalil were the negative and positive controls respectively. For each pathogen and time point, results are the mean of three replicate values ± standard deviation (SD), Figure S3: Growth of *Alternaria alternata* (panel a), *Botrytis cinerea* (panel b), *Colletotrichum acutatum sensu stricto* (*s.s.*) (panel c) and *Coniella granati* (panel d) for 72 h at 24 (±1) °C in the dark in presence of different concentrations of ellagic acid ellagic acid (EA50, EA100, EA200 mg/mL). H_2_O and imazalil were the negative and positive control, respectively. For each pathogen and time point, results are the mean of three replicate values ± standard deviation (SD), Figure S4: Growth of *Alternaria alternata* (panel a), *Botrytis cinerea* (panel b), *Colletotrichum acutatum sensu stricto* (*s.s.*) (panel c) and *Coniella granati* (panel d) for 72 h at 24 (±1) °C in the dark in the presence of different concentrations of gallic acid (GA100, GA200, GA400 mg/mL). H_2_O and imazalil were the negative and positive control, respectively. For each pathogen and time point, results are the mean of three replicate values ± standard deviation (SD), Figure S5: Molecular conformations considered as representative queries for punicalagin in the performed 3D ligand-based analyses. Panels a and b report the conformation of punicalagin deprotonated at the 3-hydroxyl (punicalagin 3O) and 8-hydroxyl (punicalagin 8O) groups of the ellagic acid moiety, respectively. The image was created with PyMol (The PyMOL Molecular Graphics System, Version 1.8, Schrödinger, LLC), Figure S6: Protonation states considered for the punicalagin natural product in the present study, Table S1: Inhibition diameters displayed by punicalagin, punicalin, ellagic acid, gallic acid, fluconazole, amphotericin B and solvent (DMSO) in the agar disk diffusion assay. Data are expressed as mean ± SD (mm). Table S2: Amount (expressed as µg/mL) of punicalagin (PG), punicalin (PA), and ellagic acid (EA) in the lysates of *Candida albicans* strains after different incubation time.
