# Supplementary material for: Antifungal Activity and DNA Topoisomerase Inhibition of Hydrolysable Tannins from Punica granatum L
Source: Int J Mol Sci. 2021 Apr 17;22(8):4175. doi: 10.3390/ijms22084175 (PMC8073005; doi:10.3390/ijms22084175)
Supplement: Supplementary file 1 [file ijms-22-04175-s001.zip › ijms-1161787-SI/Supplementary material-FIGURES MERGED.pdf]

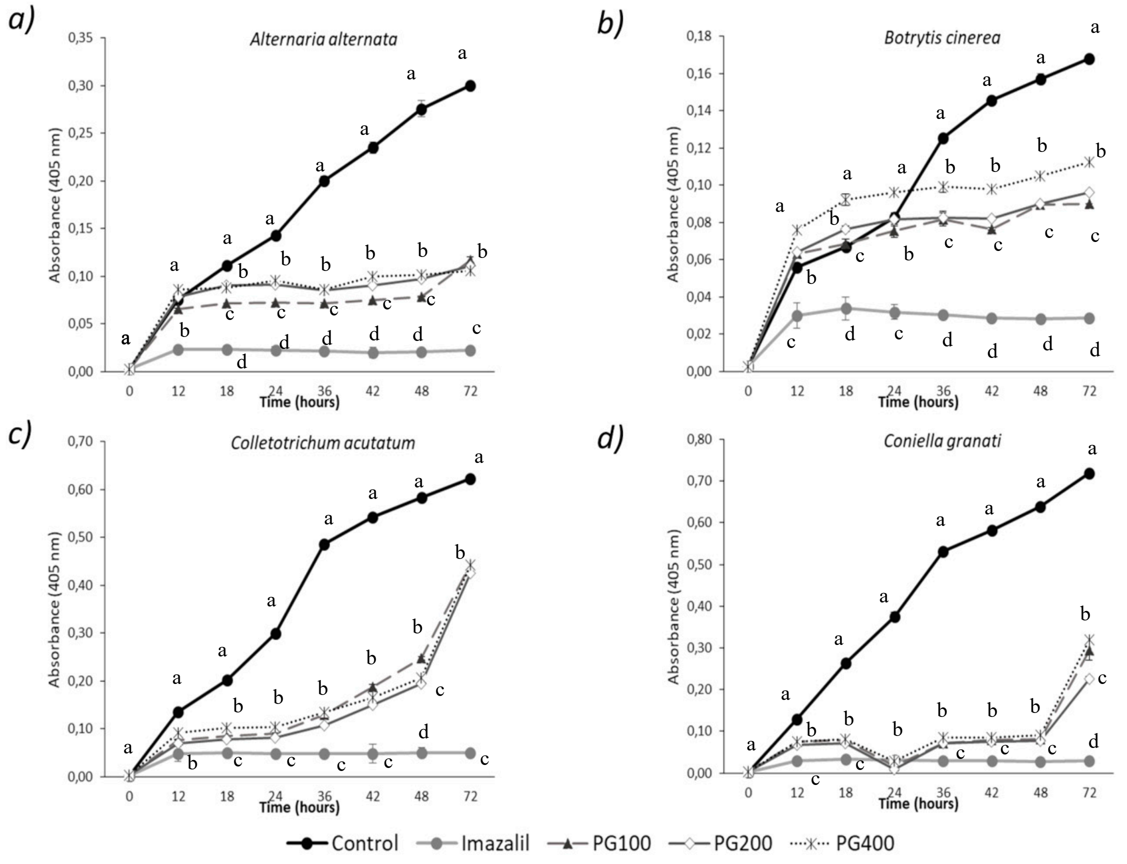

Figure S1. Growth of *Alternaria alternata* (**panel a**), *Botrytis cinerea* (**panel b**), *Colletotrichum acutatum sensu stricto* (*s.s.*) (**panel c**) and *Coniella granati* (**panel d**) for 72 h at 24 (±1) °C in the dark in presence of different concentrations of punicalagin (PG100, PG200, PG400 mg/mL). H_2O and imazalil were the negative and positive control, respectively. For each pathogen and time point, results are the mean of three replicate values ± standard deviation (SD), and different letters indicate statistical significance ($p \leq 0.05$), according to DMRT.

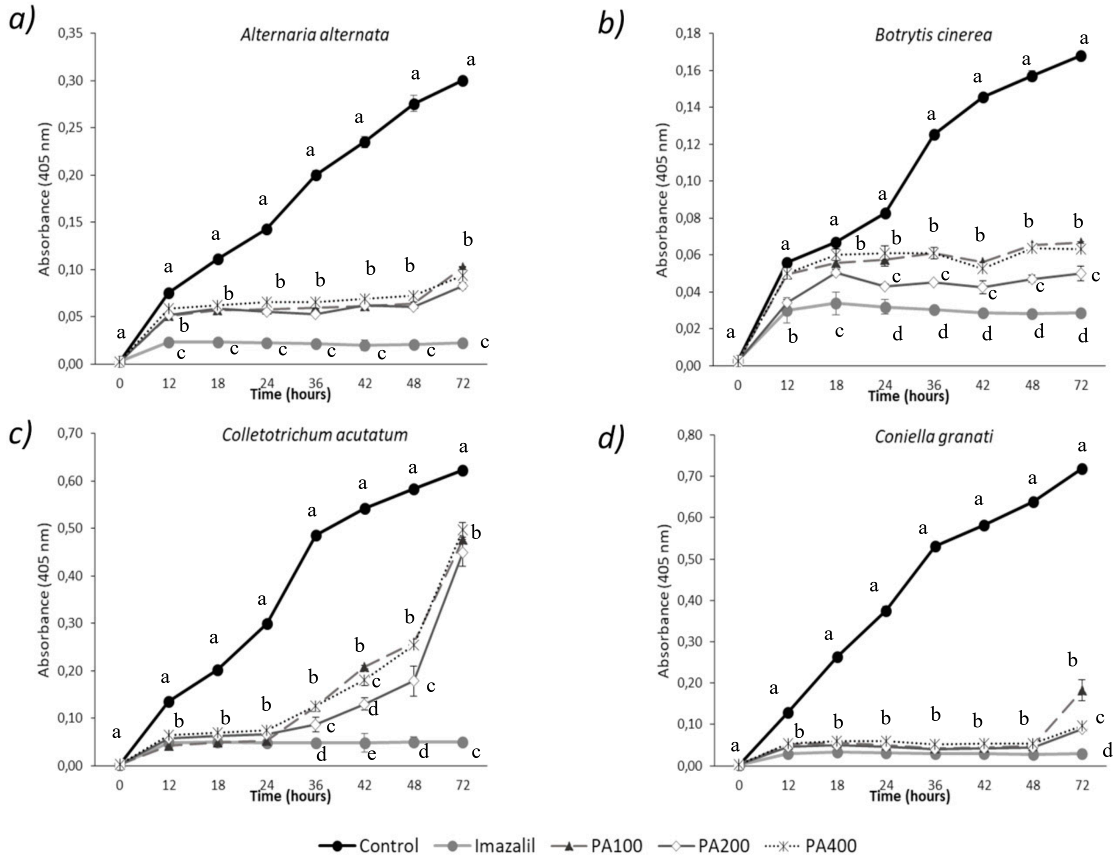

Figure S2. Growth of *Alternaria alternata* (**panel a**), *Botrytis cinerea* (**panel b**), *Colletotrichum acutatum sensu stricto* (*s.s.*) (**panel c**) and *Coniella granati* (**panel d**) for 72 h at 24 (±1) °C in the dark in presence of different concentrations of punicalin (PA100, PA200, PA400 mg/mL). H_2O and imazalil were the negative and positive control, respectively. For each pathogen and time point, results are the mean of three replicate values ± standard deviation (SD), and different letters indicate statistical significance ($p \leq 0.05$) according to DMRT.

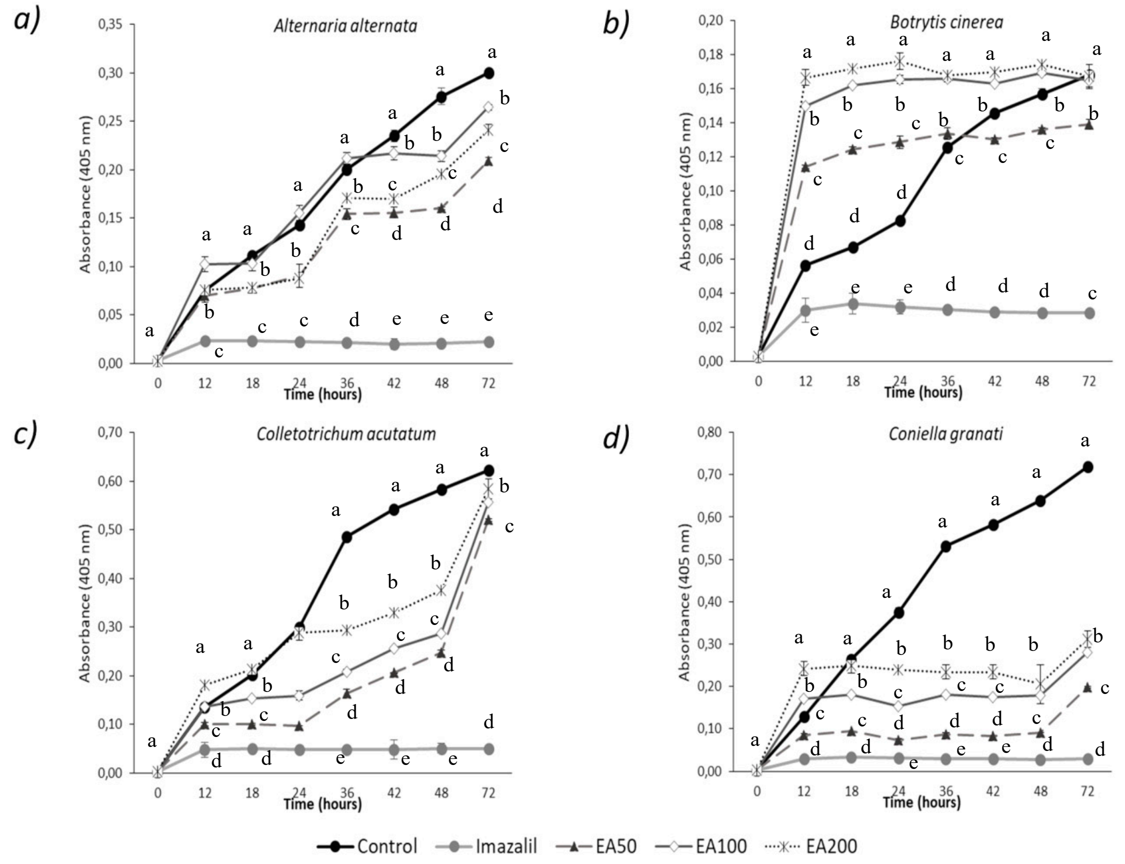

Figure S3. Growth of *Alternaria alternata* (**panel a**), *Botrytis cinerea* (**panel b**), *Colletotrichum acutatum sensu stricto* (*s.s.*) (**panel c**) and *Coniella granati* (**panel d**) for 72 h at 24 (±1) °C in the dark in presence of different concentrations of ellagic acid (EA50, EA100, EA200 mg/mL). Control (H_2O) and imazalil were the negative and positive control, respectively. For each pathogen and time point, results are the mean of three replicate values ± standard deviation (SD), and different letters indicate statistical significance ($p \leq 0.05$) according to DMRT.

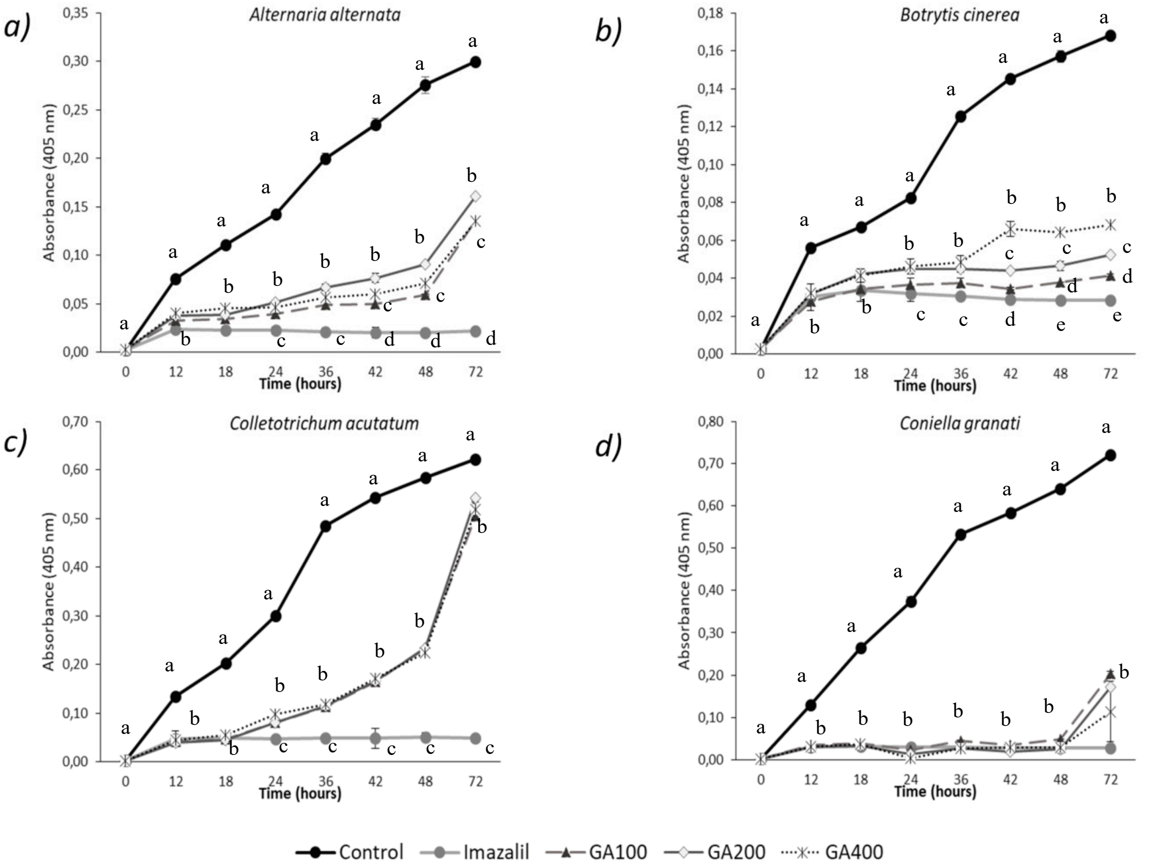

Figure S4. Growth of *Alternaria alternata* (**panel a**), *Botrytis cinerea* (**panel b**), *Colletotrichum acutatum sensu stricto* (*s.s.*) (**panel c**) and *Coniella granati* (**panel d**) for 72 h at 24 (± 1) °C in the dark in the presence of different concentrations of gallic acid (GA100, GA200, GA400 mg/mL). H_2O and imazalil were the negative and positive control, respectively. For each pathogen and time point, results are the mean of three replicate values ± standard deviation (SD), and different letters indicate statistical significance ($p \leq 0.05$) according to DMRT.

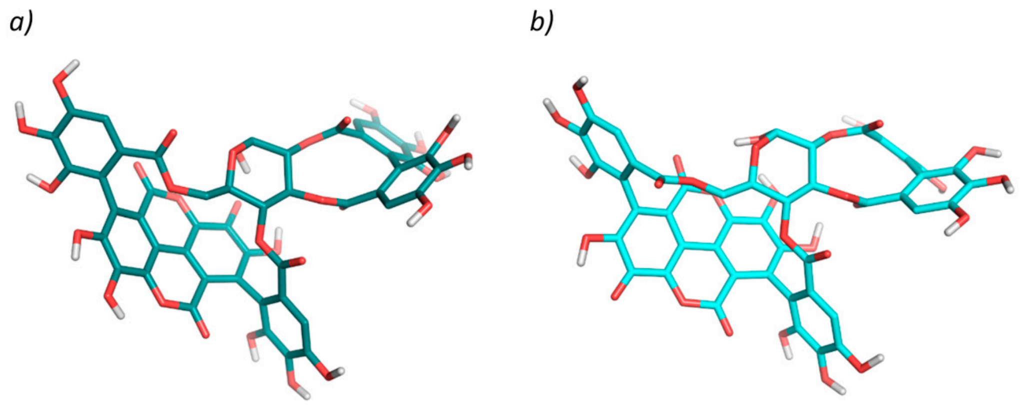

Figure S5. Molecular conformations considered as representative queries for punicalagin in the performed 3D ligand-based analyses. **Panels a** and **b** report the conformation of punicalagin deprotonated at the 3-hydroxyl (punicalagin 3O) and 8-hydroxyl (punicalagin 8O) groups of the ellagic acid moiety, respectively. The image was created with PyMol (The PyMOL Molecular Graphics System, Version 1.8, Schrödinger, LLC).

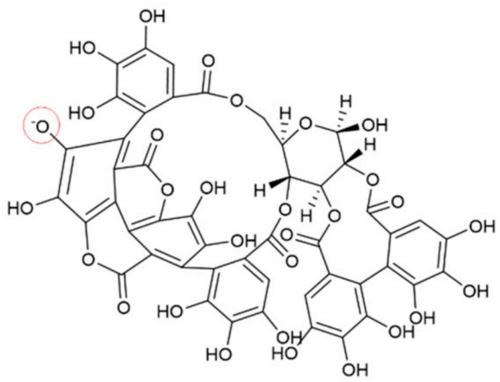

Punicalagin 3O

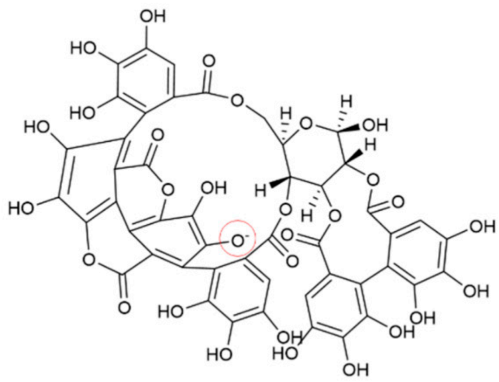

Punicalagin 8O

Figure S6. Protonation states considered for the punicalagin natural product in the present study.
