# Supplementary material for: Antifungal Activity and DNA Topoisomerase Inhibition of Hydrolysable Tannins from Punica granatum L
Source: Int J Mol Sci. 2021 Apr 17;22(8):4175. doi: 10.3390/ijms22084175 (PMC8073005; doi:10.3390/ijms22084175)
Supplement: Supplementary file 1 [file ijms-22-04175-s001.zip › ijms-1161787-SI/Supplementary material-TABLES MERGED.pdf]

Table S1

Inhibition diameters displayed by punicalagin, punicalin, ellagic acid, gallic acid, fluconazole, amphotericin B and solvent (DMSO) in the agar disk diffusion assay. Data are expressed as mean ± SD (mm).

	Punicalagin			Punicalin			Ellagic acid			Gallic acid			Fluconazole	Amphotericin B	DMSO
	50 µg	100 µg	250 µg	50 µg	100 µg	250 µg	12.5 µg	25 µg	62.5 µg	50 µg	100 µg	250 µg	25 µg	25 µg	20 µL
Candida albicans ATCC 10321	19[a]	27±0.2	35[a]	23[a]	25±0.2	32±0.1	18[a]	25±0.3	32±0.1	15[a]	21±0.2	28±0.1	22±0.2	24[a]	0[a]
Candida albicans SC 5314	21±0.3	25±0.1	34±0.3	10±0.5	16±0.5	23±0.3	16[a]	27[a]	34[a]	18±0.2	20[a]	25[a]	26±0.3	24±0.3	0[a]
Candida albicans 1	18[a]	21[a]	30[a]	15[a]	16[a]	21[a]	15±0.4	23[a]	29[a]	14[a]	19±0.1	24±0.4	24±0.5	16±0.5	0[a]
Candida albicans 2	19±0.3	21[a]	28[a]	15±0.1	18[a]	24±0.2	18[a]	22[a]	30±0.3	19[a]	25±0.1	27[a]	21±0.1	18±0.1	0[a]
Candida albicans H	20[a]	24[a]	31[a]	13±0.3	15±0.1	21±0.6	15[a]	20[a]	27[a]	15±0.4	18[a]	22±0.3	20±0.3	16±0.1	0[a]
Candida albicans 40	15[a]	20±0.4	30[a]	16±0.3	16±0.4	25[a]	17[a]	21[a]	28±0.3	14[a]	20±0.3	24[a]	15±0.5	21[a]	0[a]
Candida albicans 41	15[a]	21[a]	30[a]	18[a]	18[a]	23[a]	18±0.2	23±0.3	29±0.1	19[a]	23[a]	26[a]	14±0.5	18±0.6	0[a]
Candida albicans 44	18[a]	23±0.4	29±0.5	16[a]	18[a]	27±0.1	19[a]	23[a]	32[a]	15[a]	19±0.4	28[a]	25±0.2	16±0.2	0[a]
Candida parapsilosis	19±0.4	21±0.1	29±0.7	16±0.2	18±0.4	23[a]	15±0.5	19±0.5	26[a]	15[a]	20±0.2	23±0.1	28[a]	21±0.2	0[a]
Candida parapsilosis 7	15[a]	19[a]	28[a]	15±0.1	16[a]	25[a]	13[a]	18[a]	25[a]	14±0.1	19[a]	25±0.4	25±0.3	20±0.3	0[a]
Candida zeylanoides 33	18[a]	22±0.2	26[a]	18±0.3	18[a]	25[a]	18±0.2	20[a]	25±0.4	13[a]	20[a]	24[a]	25[a]	22[a]	0[a]
Saccharomyces cerevisiae 42	13[a]	28[a]	32[a]	20±0.5	20±0.3	23±0.3	15[a]	19[a]	23±0.2	15±0.1	18±0.2	22±0.1	30±0.6	29±0.5	0[a]
Aspergillus brasiliensis ATCC 16404	15±0.4	23[a]	28[a]	18±0.3	21[a]	25[a]	15±0.1	21[a]	25[a]	13±0.4	23[a]	29±0.2	27[a]	26±0.5	0[a]
Aspergillus candidus 3	15[a]	21[a]	25[a]	15[a]	20[a]	26[a]	15±0.4	19±0.3	25[a]	16±0.2	24[a]	27[a]	25±0.5	18±0.7	0[a]
Aspergillus candidus 25	18±0.2	20[a]	28[a]	15±0.4	18±0.1	23[a]	13±0.2	17[a]	21±0.1	15[a]	23±0.4	28[a]	27±0.2	16±0.2	0[a]
Cryptococcus neoformans B3501	15[a]	21±0.1	28[a]	21[a]	23±0.1	27±0.4	17[a]	19±0.1	24[a]	18±0.5	25[a]	32±0.1	37[a]	22[a]	0[a]
Cryptococcus neoformans ATCC 11240	15[a]	18[a]	25±0.3	18±0.1	21[a]	25[a]	15[a]	17±0.3	25[a]	15[a]	29±0.3	31±0.3	37±0.3	24[a]	0[a]
Cryptococcus 67	15±0.5	18±0.4	28[a]	16±0.3	18±0.3	23±0.1	13±0.3	18[a]	21±0.3	13±0.3	19[a]	28±0.5	31±0.1	20±0.5	0[a]
Cryptococcus var. grubii H99 serotype A	13±0.3	21[a]	28±0.2	15±0.1	20±0.3	27±0.1	15[a]	18±0.2	23[a]	15±0.3	26[a]	30[a]	35±0.2	24[a]	0[a]

[a] SD < 0.05.

Table S2

Amount (expressed as μg/mL) of punicalagin (PG), punicalin (PA) and ellagic acid (EA) in the lysates of *Candida albicans* strains at different incubation times.

	C. albicans SC5314						*C. albicans* 1					
	Punicalagin			Punicalin			Punicalagin			Punicalin		
Time (h)	PG	PA	EA	PG	PA	EA	PG	PA	EA	PG	PA	EA
12	0.72±0.11	0.05±0.02	0.02[a]	n.d.	1.55±0.04	0.02[a]	0.33±0.08	0.06±0.01	0.03[a]	n.d.	1.28±0.05	0.02[a]
24	0.95±0.02	0.10±0.01	0.03[a]	n.d.	1.56±0.13	0.02[a]	0.72±0.02	0.09±0.01	0.03[a]	n.d.	1.63±0.01	0.02[a]
48	0.58±0.02	0.09±0.01	0.04[a]	n.d.	0.95±0.18	0.02[a]	0.03 [a]	0.43±0.01	0.02[a]	n.d.	0.39±0.02	0.02[a]
72	0.62±0.06	0.07±0.01	0.03[a]	n.d.	0.64±0.09	0.01[a]	0.50±0.04	0.08±0.01	0.04[a]	n.d.	0.81±0.05	0.01[a]

[a] SD < 0.005.
